# Postpartum Triple Allograft Injury with Atypical Hemolytic Uremic Syndrome, Recurrent IgA Nephropathy, and Antibody-Mediated Rejection in a Kidney Retransplant Recipient: A Case Report

**DOI:** 10.3390/jcm15207976

**Published:** 2026-10-15

**Authors:** Xuelian Chen, Dongmei Wang, Lichuan Yang, Li Qiu, Jiaojiao Zhou

**Affiliations:** 1 Department of Medical Ultrasound, West China Hospital, Sichuan University, Chengdu 610041, China; 2023324020058@stu.scu.edu.cn (X.C.); wdongm11@wchscu.cn (D.W.); 2 Department of Nephrology, West China Hospital, Sichuan University, Chengdu 610041, China; ylcgh@163.com

**Keywords:** kidney transplantation, second kidney transplantation, pregnancy, atypical hemolytic uremic syndrome, recurrent IgA nephropathy, antibody-mediated rejection

## Abstract

**Background:** The concurrent presentation of chronic hypertension with superimposed preeclampsia, postpartum atypical hemolytic uremic syndrome (aHUS), recurrent IgA nephropathy (IgAN), and antibody-mediated rejection (ABMR) constitutes a life-threatening clinical emergency. To the best of our knowledge, the coexistence of all these complications has rarely been documented in the literature, representing an extremely rare clinical phenotype. **Methods**: This study presents a 33-year-old female who had undergone two prior living-donor kidney transplantations. She underwent emergency cesarean section at 32 weeks of gestation due to threatened uterine rupture superimposed with preeclampsia. Postoperatively, allograft function deteriorated rapidly. Renal allograft biopsy confirmed triple pathological lesions including recurrent IgAN, aHUS, and ABMR. Genetic testing detected no pathogenic mutations classically associated with aHUS. **Results:** The patient received targeted complement inhibition with eculizumab combined with intensified anti-rejection therapy and renal replacement therapy. Hemolysis-related parameters showed only transient improvement while the condition progressed continuously. The patient eventually died of multiple organ failure five months postpartum. **Conclusions**: This case highlights the need to differentiate multiple concurrent etiologies in second living-donor kidney transplant recipients with post-pregnancy renal decline. Thrombotic microangiopathy, recurrent primary renal disease, and allograft rejection should be suspected in postpartum patients with renal injury, thrombocytopenia, and anemia. Despite early biopsy and combination therapy, this severe complication constellation has a dismal prognosis and can cause fatal multiple organ failure, reflecting diagnostic challenges and therapeutic limitations in this rare presentation.

## 1. Introduction

Obstetricians and nephrologists frequently encounter clinical challenges regarding pregnancy in kidney transplant recipients [1]. Pregnancy after kidney transplantation represents a high-risk clinical scenario, associated with substantially elevated risks of pre-eclampsia, allograft loss, and adverse maternal–fetal outcomes [2,3,4]. Atypical hemolytic uremic syndrome (aHUS) is characterized by the classic triad of microangiopathic hemolytic anemia, thrombocytopenia, and acute kidney injury (AKI), driven by dysregulated activation of the alternative complement pathway [5]. Pregnancy constitutes an important trigger for aHUS, Pregnancy-related hemodynamic shifts, endothelial injury, and hypertensive disorders of pregnancy may induce secondary thrombotic microangiopathy [6,7]. Pregnancy-associated aHUS occurs predominantly in the postpartum period, frequently following various gestational complications [7].

Recipients with repeated kidney retransplants have a higher risk of allograft failure and rejection compared with patients receiving a single retransplant [8]. Furthermore, the risk of rejection rises markedly during pregnancy and the postpartum period [9]. Recipients with prior IgA nephropathy (IgAN) recurrence who undergo repeat living-donor kidney transplantation carry dual risks of recurrent primary glomerulopathy and allo-sensitization. Physiological stress imposed by pregnancy may superimpose multiple renal insults on this high-risk background [10,11]. For recipients whose original disease was IgAN, both pre-pregnancy transplant-to-conception interval are independent correlates of gestational hypertension. Moreover, transplant allograft survival is poorer in pregnant recipients with underlying IgAN relative to those with other primary renal diseases. Accordingly, kidney transplant recipients should be counseled about the risks of gestational hypertension and potential long-term deterioration of renal function after delivery [12]. 

The simultaneous co-occurrence of postpartum aHUS, recurrent primary glomerular disease, and antibody-mediated rejection is exceedingly rare. Since kidney injury serves as a major shared manifestation of these three conditions, clinical misdiagnosis is highly likely. Delayed recognition of multi-etiological renal injury may lead to inappropriate management and poor prognosis. Herein, we report a rare case of triple renal injury following pregnancy in a recipient of a second living-donor kidney transplant, and summarize the diagnostic challenges and therapeutic considerations under this complex clinical circumstance.

## 2. Case Presentation

A 33-year-old female presented with proteinuria for one month more than 10 years after kidney transplantation. More than 10 years ago, she received her first living-donor kidney transplant from her father for end-stage renal disease secondary to IgAN. Allograft function remained stable in the early post-transplant period. Proteinuria and progressive renal impairment developed more than seven years later. Recurrent IgAN was confirmed by allograft biopsy. She then initiated maintenance hemodialysis due to graft loss. More than three years before current admission, she underwent a second living-donor kidney transplantation with a graft from her mother. Post-transplant allograft function was stable. Approximately 10 months prior to admission, mycophenolate sodium was switched to azathioprine for pregnancy preparation. A singleton intrauterine pregnancy was confirmed eight months before admission. 

**Figure 1:**
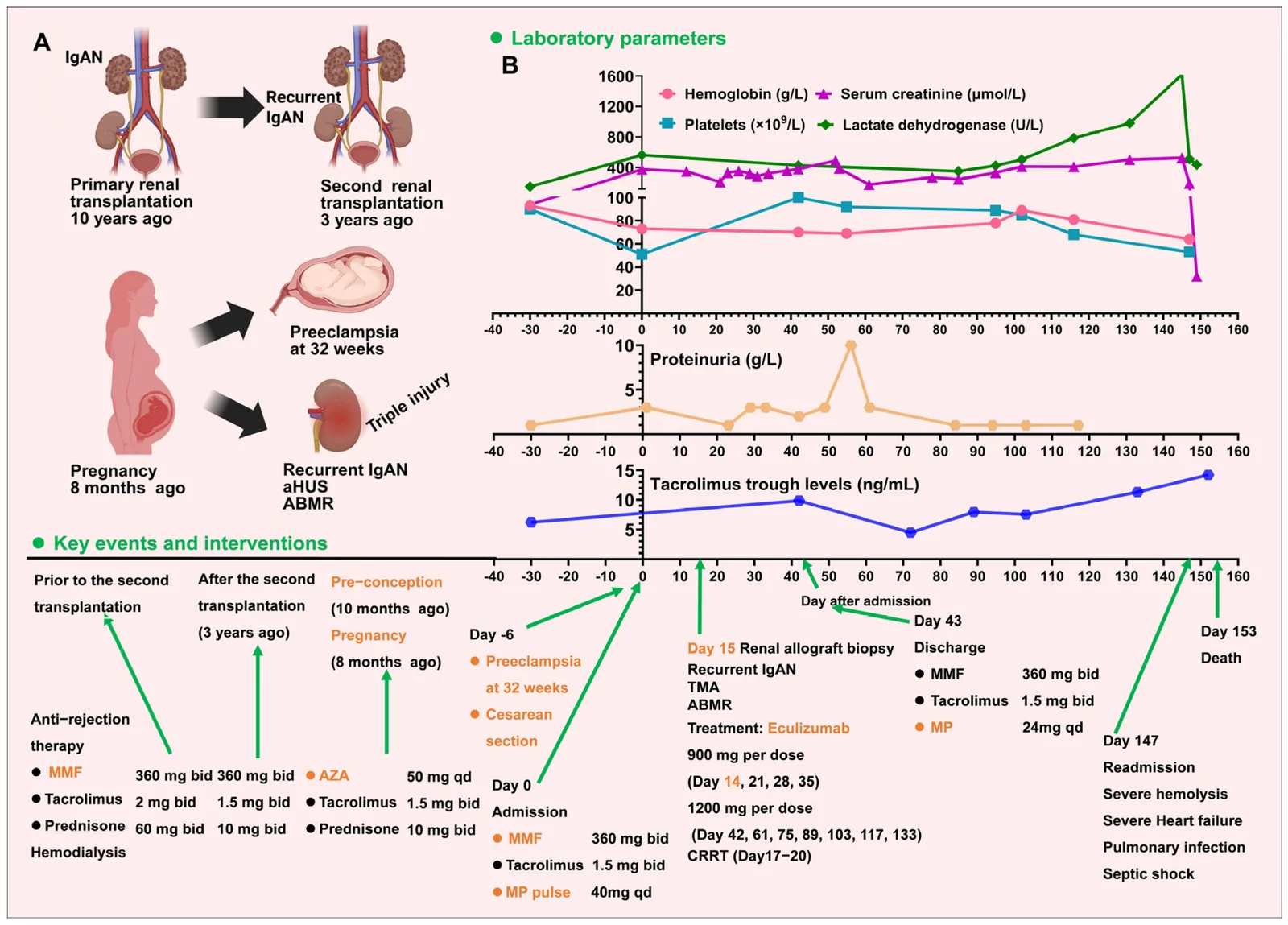
Clinical course and interventions of postpartum renal allograft dysfunction in a patient with secondary kidney transplantation. (**A**) Schematic diagram of the Clinical course. (**B**) Longitudinal monitoring of laboratory parameters and key clinical events along the time axis (Day 0 = day of index admission). Abbreviations: IgA Nephropathy, IgAN; ABMR, antibody-mediated rejection; aHUS, atypical hemolytic uremic syndrome; MP, methylprednisolone; MMF, mycophenolate mofetil; AZA, azathioprine; TMA, thrombotic microangiopathy; CRRT, continuous renal replacement therapy.

One month prior to admission, she developed proteinuria accompanied by anemia and thrombocytopenia. Six days before admission (gestational week 32^+1^), she presented with acute abdominal pain. She was diagnosed with threatened uterine rupture, preeclampsia superimposed on chronic hypertension, thrombocytopenia and preterm labor at an outside hospital and underwent emergency cesarean section. Severe oliguria occurred after delivery. Her serum creatinine rose rapidly to 410 μmol/L. She was admitted to the intensive care unit for continuous renal replacement therapy (CRRT), blood transfusion, anti-infective and other supportive care and later transferred to the nephrology department of our hospital for further evaluation and management. The detailed clinical course is illustrated in Figure 1.

Laboratory and imaging findings are as follows: hemoglobin 70 g/L (Reference range: 115–150 g/L), Platelets 67 × 10^9^/L (Reference range:100–300 × 10^9^/L), serum creatinine 376 μmol/L (Reference range: 48–79 μmol/L), lactate dehydrogenase 563 IU/L (Reference range: 120–250 IU/L), albumin 34.1 g/L (Reference range: 40–55 g/L), proteinuria (+++). 

HLA antibodies (−), Non-HLA antibodies: anti-CSF2 (+++), anti-PRKCH (++), Thyroglobulin (+), anti-ANA antibodies (−), anti-ds-DNA antibodies (−), anti-ENA antibodies (−), anti-CCP antibody (−), anti-GBM antibody (−), anti-PLA2R antibody (−). Red blood cell anisocytosis 19.6% (Reference range: <11%), schistocyte percentage 0.6% (Reference: 0%), C5b-9 359 ng/mL (Reference range: 72–252 ng/mL), C3 0.612 g/L (Reference range: 0.7–1.4 g/L), C4 0.17 g/L (Reference range: 0.1–0.4 g/L). ADAMTS13 activity 58.8% (Reference range: >10%), anti-CFH antibody (−). aHUS-related genetic testing (−). 

**Figure 2:**
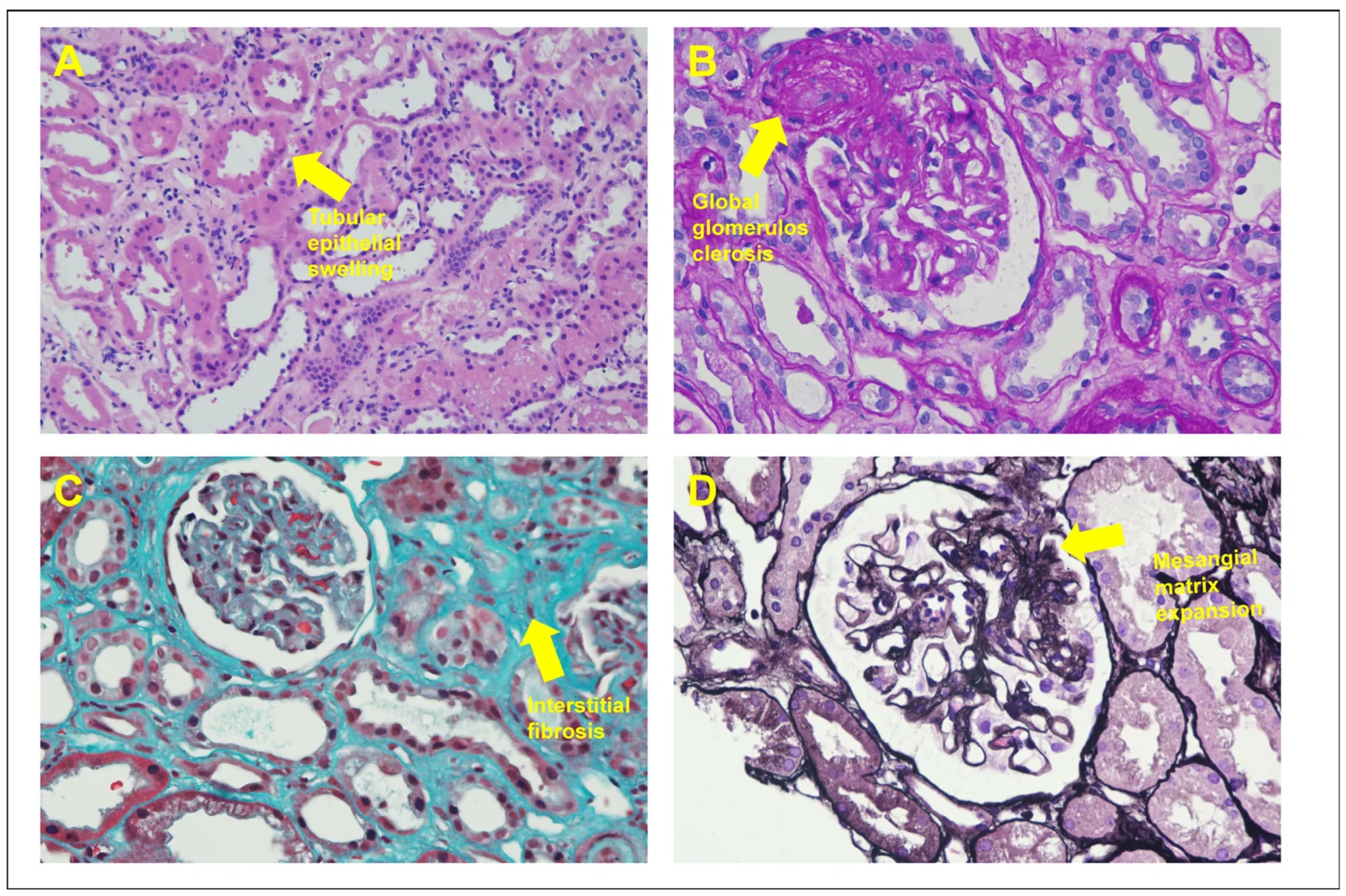
Light microscopic pathological findings of renal allograft biopsy. (**A**) Hematoxylin-eosin (HE) staining, ×200. (**B**) Periodic acid-Schiff (PAS) staining, ×400. (**C**) Masson’s trichrome staining, ×400. (**D**) Periodic acid-silver methenamine (PASM) staining, ×400.

**Figure 3:**
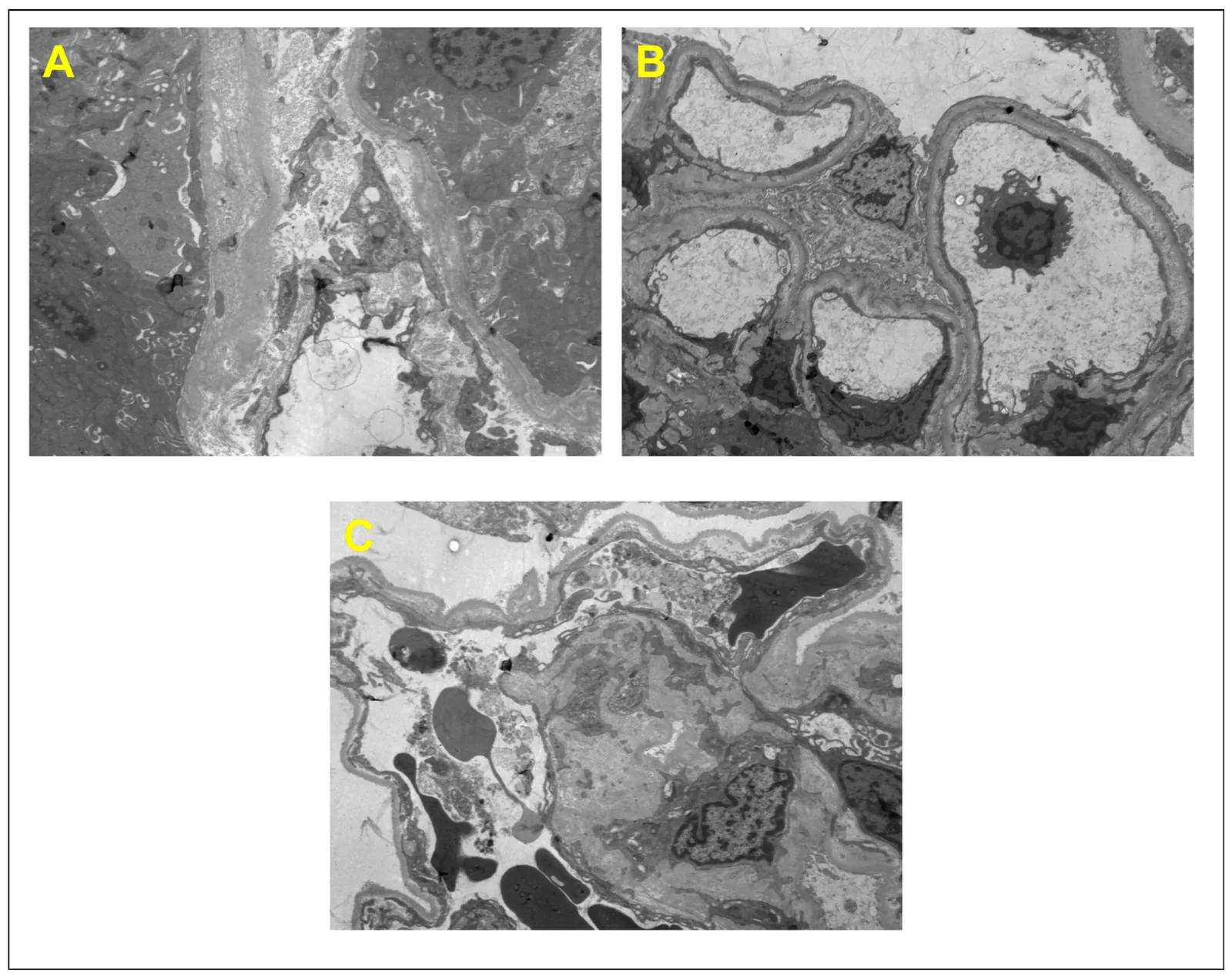
Ultrastructural features of renal allograft biopsy (transmission electron microscopy). (**A**) Focal mild-to-moderate mesangial expansion with mesangial cell and matrix proliferation and segmental subendothelial edema and glomerular basement membrane thickening. ×3000 magnification. (**B**) Segmental capillary luminal collapse, endothelial cell necrosis, mild-to-moderate neutrophil exudation, and segmental mesangial interposition. ×2000 magnification. (**C**) Multisegmental podocyte foot process effacement, microvillous transformation, segmental foot process necrosis and GBM denudation. ×2500 magnification.

Renal allograft Doppler ultrasonography revealed allograft hydronephrosis (1.2 cm), elevated resistance index (RI) of main renal artery (0.82) and segmental arteries (0.77), and small volume of intra-abdominal effusion [13]. Renal tissue samples were collected by ultrasound-guided percutaneous renal biopsy on Day 15, with pathological diagnoses summarized in Table 1 and representative histopathological images shown in Figure 2 and Figure 3.

## 3. Diagnosis, Management and Clinical Outcome

The patient received a clinical diagnosis of triple overlapping pathology including postpartum aHUS, recurrent IgAN, and ABMR. Eculizumab was initiated as targeted complement inhibition therapy with a 900 mg induction dose administered once weekly on Day 14, 21, 28, and 35. This was followed by 1200 mg maintenance doses delivered on Day 42, 61, 75, 89, 103, 117, and 133, bringing the total cumulative dose to 12 g. Concurrently, the patient received methylprednisolone pulse therapy plus intensified immunosuppression with tacrolimus and mycophenolate sodium. Additional comprehensive supportive measures included continuous renal replacement therapy (CRRT), blood product transfusion, antihypertensive agents, and anti-infective treatment.

After the above interventions, the patient showed improvements in hemoglobin, platelet count, and hemolysis-related biomarkers, but serum creatinine remained persistently elevated. The patient’s clinical condition later deteriorated again and was complicated by severe hemolysis, severe heart failure, pulmonary infection, and septic shock. Despite rescue therapy including plasma exchange, mechanical ventilation, ongoing CRRT, and anti-infective agents, the patient succumbed to multiple organ failure on Day 153. The patient’s family withheld consent for autopsy, so the precise cause of death could not be further confirmed.

## 4. Discussion

AKI associated with pregnancy can occur during all trimesters and the postpartum period. Etiologies include insufficient renal perfusion, pre-eclampsia, immune-mediated injury and other factors. It represents a major global perinatal health burden associated with markedly increased maternal-fetal morbidity and mortality [18]. Its pathophysiology involves decreased renal perfusion, endothelial dysfunction and inflammatory cascade activation, which provide the pathological substrate for the development of thrombotic microangiopathy [19]. Pregnancy in kidney transplant recipients requires multidisciplinary whole-course management by nephrologists, midwives and high-risk obstetric specialists [20]. Standardized antenatal monitoring enables early recognition and timely intervention for complications such as hypertension, pre-eclampsia and allograft dysfunction, and the risk of adverse outcomes is directly correlated with the degree of pre-pregnancy renal impairment [21]. Although kidney transplant recipients have better perinatal outcomes compared with women on dialysis, the risks of adverse pregnancy events and renal injury remain substantially elevated even among those with stable renal function [22]. The increased risk of pregnancy-related renal injury in the setting of chronic kidney disease and kidney transplantation is closely related to baseline renal function, immunosuppressive status and underlying primary renal disease [4,9]. Pregnancies in solid-organ transplant recipients are associated with a 4- to 6-fold higher risk of pre-eclampsia, preterm birth and low birth weight, whereas the overall risk of allograft rejection or graft loss during pregnancy is low [23]. 

This case presents a triad of allograft injury comprising aHUS-phenotype thrombotic microangiopathy, recurrent IgAN, and C4d-positive antibody-mediated rejection. These three processes are not a coincidental co-occurrence of independent diseases; rather, against the dual high-risk background of kidney retransplantation and pregnancy, they share a well-defined susceptibility basis and a pathological cascade-amplification effect that collectively drives rapidly progressive allograft injury. Kidney retransplantation itself constitutes a high-risk predisposing substrate for the coexistence of this triple allograft injury. First, the patient developed allograft failure secondary to recurrent IgAN following her first kidney transplantation, indicating an intrinsic predisposition to IgA-related mucosal immune dysregulation. Accumulating evidence has demonstrated that kidney transplant recipients with primary IgAN carry significantly elevated risks of IgAN recurrence, allograft injury, and obstetric complications during pregnancy [12]. The risk of allograft loss is inherently higher in kidney retransplant recipients than in first-time transplant recipients [8]. Precision risk stratification is warranted for patients with high-recurrence kidney diseases undergoing living-donor kidney transplantation, with reproductive intent serving as an important risk-modifying factor [10]. Secondly, re-transplant recipients typically exhibit a more prominent pre-transplant sensitization status. To avoid the teratogenic risk of mycophenolate sodium during the preconception period, it is frequently replaced with azathioprine. This therapeutic adjustment may reduce the overall intensity of immunosuppression, which could theoretically increase the risk of antibody-mediated rejection and potentially attenuate the inhibitory effect on mesangial IgA deposition [1,20,24]. Third, profound maternal physiological perturbations take place during the peripartum period, including physiological complement activation, elevated vascular endothelial stress, and heightened neutrophil activation. This period is well-recognized as a trigger window for thrombotic microangiopathy [6,7,25,26,27]. Epidemiological data demonstrate that pregnancy-associated aHUS occurs in approximately 0.02% of all pregnancies. In contrast, the overall incidence of thrombotic microangiopathy among kidney transplant recipients ranges from 0.8% to 14%, which is substantially higher than that in the general population [5,28]. 

For patients with kidney retransplantation and reproductive intent, clinicians face a core clinical dilemma of balancing the teratogenic risk of potent immunosuppressants against the high risks of primary disease recurrence, allosensitization and allograft rejection [29]. While preconception conversion from mycophenolate sodium to azathioprine, as recommended by international guidelines [30], reduces fetal teratogenic exposure, mycophenolate mofetil has demonstrated superiority over azathioprine in improving graft survival and preventing acute rejection after kidney transplantation [31]. This conversion may therefore lead to subtherapeutic immunosuppression, which in turn elevates the risks of rejection and IgAN recurrence, and constitutes an important predisposing factor for the triple allograft injury observed in this case. Individualized risk stratification, full informed consent on both maternal and fetal outcomes, and tailored immunosuppressive regimens are warranted for retransplant recipients with high sensitization and high recurrence risk who plan pregnancy [12]. 

Thrombotic microangiopathy (TMA) in postpartum kidney transplant recipients presents a substantial diagnostic challenge, as clinical manifestations, laboratory profiles and histopathological features overlap extensively across different etiologies. Thrombotic thrombocytopenic purpura (TTP) was first ruled out. The gold-standard diagnostic cutoff for TTP is ADAMTS13 activity < 10%, and the disease typically manifests with severe thrombocytopenia and neurological involvement [32]. HELLP syndrome and severe preeclampsia-related TMA typically present with elevated liver transaminases, microangiopathic hemolysis and normal ADAMTS13 activity. However, hemolysis and end-organ damage attributable to preeclampsia generally resolve within 72 h after delivery, which was not the case in this patient. Calcineurin inhibitor-induced TMA usually occurs in the setting of supratherapeutic CNI trough levels [33]. The patient maintained stable tacrolimus exposure throughout the clinical course, making isolated CNI-induced TMA an unlikely explanation. Complement profiling revealed decreased C3, normal C4, and markedly elevated terminal complement complex C5b-9 in this patient. While complement activation has been documented in preeclampsia and HELLP syndrome, both of which are microangiopathies of late pregnancy [34], the pattern and magnitude of complement dysregulation here were consistent with a more pronounced complement-mediated pathological process rather than transient physiological activation of pregnancy. Taken together, the overall clinical and laboratory findings were most consistent with pregnancy-associated TMA with a suspected complement-mediated aHUS phenotype rather than unmasked hereditary aHUS. Given that terminal complement pathway activation mediates microvascular injury, eculizumab therapy was initiated to selectively inhibit C5 cleavage, thereby blocking complement-mediated endothelial damage and arresting the progression of thrombotic microangiopathy [6,7].

The complement activation observed in this case may not arise from an intrinsic complement regulatory defect, but rather represents secondary complement activation and subsequent thrombotic microangiopathy driven by the combined effects of antibody-mediated rejection, recurrent IgAN, and physiological changes of pregnancy. The temporospatial overlap of these three high-risk factors in a single patient may simultaneously trigger three distinct pathological processes, which form a mutually amplifying positive-feedback loop via the complement system, endothelial injury and inflammatory response, potentially exerting tissue-damaging effects far greater than those induced by any single disorder [35]. Donor-specific antibodies may bind endothelial antigens such as HLA molecules, initiating complement activation that culminates in membrane attack complex (MAC) formation. These complexes may disrupt endothelial cell membranes, induce cell lysis and compromise vascular integrity, further promoting platelet aggregation and microvascular thrombosis, impairing blood perfusion and oxygen supply within the renal allograft, and ultimately contributing to TMA development and allograft failure [25,26,28,36]. Interaction between mesangial IgA-containing immune complexes and glomerular capillary walls, combined with dysregulated complement control and C4b deposition, may also promote TMA lesion formation [12,37]. Excessive neutrophil activation and increased release of neutrophil extracellular traps during pregnancy may act synergistically with complement activation to further aggravate microvascular endothelial injury and thrombosis [25]. Antibody-mediated rejection and IgAN activate the complement system via the classical and alternative pathways, respectively [26,38]. When superimposed on physiological baseline complement activation in pregnancy [39], the overall complement-activating effect may exceed that induced by isolated aHUS. Furthermore, the therapeutic value of C5 inhibitors in ABMR-associated TMA remains unproven [40]. This may explain why this patient rapidly progressed to irreversible multi-organ failure and subsequent death.

## 5. Conclusions

We report a second living-donor kidney transplant recipient with fatal combined renal injury from concurrent postpartum aHUS, recurrent IgAN, and ABMR, in the setting of pregnancy complicated by chronic hypertension and preeclampsia. Given the highly overlapping clinical features, postpartum thrombocytopenia and hemolytic anemia often lead to a preferential TMA diagnosis, with risk of missing concomitant IgA nephropathy relapse and ABMR. Early allograft biopsy is important for identifying mixed pathologies in transplant recipients with unexplained postpartum acute kidney injury and hematologic abnormalities. However, as a single case report, conclusions regarding interactions among these three entities remain limited, and definitive causality cannot be assigned to these overlapping pathological processes. Despite timely diagnosis and combined therapy, this multifactorial severe postpartum renal injury progressed rapidly to irreversible multiple organ failure and death, highlighting the diagnostic challenges and therapeutic limitations of this rare condition. 

## Figures and Tables

**Table 1:** Pathological findings and diagnostic basis of the renal allograft biopsy.

Pathological Diagnosis	Light Microscopic Morphological Findings	Immunofluorescence /Immunohistochemical Findings	Electron Microscopic Findings	Diagnostic Criteria
IgAN	Total 23–24 glomeruli, with 3–5 globally sclerosed glomeruli; 1 fibrous crescent in Bowman’s capsule; segmental to global mesangial hypercellularity and mild matrix expansion (mm0), with 3 glomeruli showing glomerulocapsular adhesion and segmental sclerosis; segmental wrinkling of glomerular basement membrane (GBM); segmental hyaline thrombus in 1 glomerulus, with segmental endothelial swelling and luminal occlusion; glomerulitis (g1)	Immunofluorescence: IgA (+~++) in mesangial regions of 2/3 glomeruli; λ (+) in segmental mesangial regions of 3/5 glomeruli; κ (+) in segmental mesangial regions of 2/5 glomeruli; IgM, C3, IgG, C4, C1q negative in 3–5 glomeruli (glomeruli showing ischemic changes). Immunohistochemistry: C4d positive (+, C4d2); SV40 negative; CD4 positive (majority of T cells); CD8 positive (minority of T cells); CD20 positive (B cells)	Total 2 glomeruli, both with obvious lesions; focal mild-to-moderate mesangial widening with mesangial cell proliferation and matrix increase; segmental subendothelial edema and GBM thickening; multifocal foot process effacement, microvillous transformation, segmental foot process necrosis and denudation of GBM; segmental collapse of capillary lumina, endothelial cell necrosis, mild-to-moderate neutrophil infiltration, and segmental mesangial interposition; no obvious Bowman’s capsule lesions	Predominant IgA-dominant immune complex deposition in glomerular mesangium, accompanied by mesangial proliferation, glomerulosclerosis, crescent formation and other morphological lesions. In the context of renal transplantation and prior history of primary IgAN, the findings support a diagnosis of recurrent disease [14].
TMA	Segmental hyaline thrombus in 1 glomerulus, with segmental endothelial swelling and luminal occlusion; moderate-to-severe thickening of small artery and arteriole walls with intimal swelling, focal scant lymphocyte infiltration, and focal calcification (ah0, cv0, v2)	Immunofluorescence: glomeruli showing ischemic changes. Immunohistochemistry: C4d positive (+, C4d2)	Segmental collapse of capillary lumina, endothelial cell necrosis, mild-to-moderate neutrophil infiltration, and segmental mesangial interposition; segmental subendothelial edema and GBM thickening; focal tubular atrophy and focal interstitial fibrosis. Electron microscopic diagnosis: consistent with thrombotic microangiopathy	Thrombus formation in microvessels including glomerular capillaries and renal arterioles, endothelial swelling and necrosis, vascular wall thickening with luminal stenosis, and accompanying ischemic injury to renal parenchyma. The morphological findings meet the core diagnostic features of TMA [15].
ABMR	Glomerulitis (g1); intimal swelling and focal lymphocyte infiltration in small arteries and arterioles (v2); ~5% interstitial fibrosis (ci1) with infiltration of lymphocytes, monocytes and plasma cells (i1); ~5% tubular atrophy (ct1)	Immunohistochemistry: positive peritubular capillary C4d deposition (+, C4d2); predominant CD4^+^ T cell infiltration in renal interstitium, accompanied by CD8^+^ T cells and CD20^+^ B cells; SV40 negative, ruling out polyomavirus nephropathy	Glomerular capillary endothelial swelling and necrosis, subendothelial edema and GBM thickening, and capillary luminal collapse	evidence of acute tissue injury including glomerulitis and arteritis, combined with evidence of endothelial activation (positive peritubular capillary C4d). In the clinical context of renal transplantation, the findings fulfill the pathological diagnosis of ABMR [16,17].

## Data Availability

No new data were created or analyzed in this study. Data sharing is not applicable to this article.

## Abbreviations

The following abbreviations are used in this manuscript:

aHUS	Atypical hemolytic uremic syndrome
ABMR	Antibody-mediated rejection
AKI	Acute kidney injury
CRRT	Continuous renal replacement therapy
TMA	Thrombotic microangiopathy
MP	Methylprednisolone
MMF	Mycophenolate mofetil
AZA	Azathioprine

## References

[1] Boulay H., Mazaud-Guittot S., Supervielle J., Chemouny J.M., Dardier V., Lacroix A., Dion L., Vigneau C. (2021). Maternal, foetal and child consequences of immunosuppressive drugs during pregnancy in women with organ transplant: A review. Clin. Kidney J..

[2] Bramham K., Nelson-Piercy C., Gao H., Pierce M., Bush N., Spark P., Brocklehurst P., Kurinczuk J.J., Knight M. (2013). Pregnancy in renal transplant recipients: A UK national cohort study. Clin. J. Am. Soc. Nephrol..

[3] Shah P.B., Samra M., Josephson M.A. (2018). Preeclampsia Risks in Kidney Donors and Recipients. Curr. Hypertens. Rep..

[4] Webster P., Lightstone L., McKay D.B., Josephson M.A. (2017). Pregnancy in chronic kidney disease and kidney transplantation. Kidney Int..

[5] Yan K., Desai K., Gullapalli L., Druyts E., Balijepalli C. (2020). Epidemiology of Atypical Hemolytic Uremic Syndrome: A Systematic Literature Review. Clin. Epidemiol..

[6] Fakhouri F., Scully M., Provôt F., Blasco M., Coppo P., Noris M., Paizis K., Kavanagh D., Pène F., Quezada S. (2020). Management of thrombotic microangiopathy in pregnancy and postpartum: Report from an international working group. Blood.

[7] Gupta M., Govindappagari S., Burwick R.M. (2020). Pregnancy-Associated Atypical Hemolytic Uremic Syndrome: A Systematic Review. Obstet. Gynecol..

[8] Kanbay M., Abdel-Rahman S.M., Brinza C., Ozbek L., Yayci E., Aktas O., Genc C., Guldan M., Alper E.N., Burlacu A. (2025). A meta-analysis of graft survival, patient survival and delayed graft function in first-time and repeat kidney transplants. Nephrol. Dial. Transplant..

[9] Mustafa M.S., Noorani A., Abdul Rasool A., Tashrifwala F.A.A., Jayaram S., Raja S., Jawed F., Siddiq M.U., Shivappa S.G., Hameed I. (2024). Pregnancy outcomes in renal transplant recipients: A systematic review and meta-analysis. Womens Health.

[10] Verhellen C., Devresse A., Gillion V., Van Regemorter E., Darius T., Buemi A., Goffin E., Kanaan N. (2026). Living Donor Kidney Transplantation in High-Recurrence Kidney Diseases: Precision Risk Stratification and Management. Kidney Int. Rep..

[11] Che M., Moran S.M., Smith R.J., Ren K.Y.M., Smith G.N., Shamseddin M.K., Avila-Casado C., Garland J.S. (2024). A case-based narrative review of pregnancy-associated atypical hemolytic uremic syndrome/complement-mediated thrombotic microangiopathy. Kidney Int..

[12] Oki R., Unagami K., Kakogawa J., Beppu H., Banno T., Yagisawa T., Kanzawa T., Hirai T., Omoto K., Kitajima K. (2023). Pregnancy Complications and Impact on Kidney Allograft After Kidney Transplantation in IgA Nephropathy. Transpl. Int..

[13] Guo Y., Yang Y., Luo S., Ren Y., Chen H., Yang Y., Shi S., Yang L., Li Y., Zhou J. (2025). The value of intrarenal resistive index in delayed graft function after kidney transplantation. Quant. Imaging Med. Surg..

[14] (2021). Kidney Disease: Improving Global Outcomes (KDIGO) Glomerular Diseases Work Group. KDIGO 2021 Clinical Practice Guideline for the Management of Glomerular Diseases. Kidney Int..

[15] Afrouzian M., Kozakowski N., Liapis H., Broecker V., Truong L., Avila-Casado C., Regele H., Seshan S., Ambruzs J.M., Farris A.B. (2023). Thrombotic Microangiopathy in the Renal Allograft: Results of the TMA Banff Working Group Consensus on Pathologic Diagnostic Criteria. Transpl. Int..

[16] Loupy A., Haas M., Roufosse C., Naesens M., Adam B., Afrouzian M., Akalin E., Alachkar N., Bagnasco S., Becker J.U. (2020). The Banff 2019 Kidney Meeting Report (I): Updates on and clarification of criteria for T cell- and antibody-mediated rejection. Am. J. Transpl..

[17] Carrigan I., Mathur S., Bourgeois N., Dieudé M., Fantus D., Gongal P., Halpin A., Hirji A., Mansell H., Piotrowski C. (2023). Updates in Kidney Transplantation From the 2022 Banff-Canadian Society of Transplantation Joint Meeting: Conference Report. Can. J. Kidney Health Dis..

[18] Kontomanolis E.N., Prokopakis I., Koutras A., Andreou E., Metaxas D., Boulieris G., Zachariou E., Sapantzoglou I., Papageorgiou D., Palios V.C. (2025). Pregnancy-Related Acute Kidney Injury: Causes and Its Impact on Perinatal Outcomes-A Systematic Review. J. Clin. Med..

[19] Kashani K.B., Wium L., Ostermann M., Mehta R.L., Ronco C., Nelson-Piercy C., Poli-de-Figueiredo C.E., Sahay M. (2026). Pathophysiology of pregnancy-associated acute kidney injury. Nephrol. Dial. Transplant..

[20] Chandra A., Midtvedt K., Åsberg A., Eide I.A. (2019). Immunosuppression and Reproductive Health After Kidney Transplantation. Transplantation.

[21] Ponticelli C., Zaina B., Moroni G. (2021). Planned Pregnancy in Kidney Transplantation. A Calculated Risk. J. Pers. Med..

[22] Cuthbertson L., Hewawasam E., Kitsos A., Saunder T., Jesudason S., Jose M. (2026). Impact of pre-conception kidney function on pregnancy outcomes. Nephrol. Dial. Transplant..

[23] Yo J.H., Fields N., Li W., Anderson A., Marshall S.A., Kerr P.G., Palmer K.R. (2024). Adverse Pregnancy Outcomes in Solid Organ Transplant Recipients: A Systematic Review and Meta-Analysis. JAMA Netw. Open.

[24] Chandrakantan A., Ratanapanichkich P., Said M., Barker C.V., Julian B.A. (2005). Recurrent IgA nephropathy after renal transplantation despite immunosuppressive regimens with mycophenolate mofetil. Nephrol. Dial. Transplant..

[25] Zaimi M., Kambas K., Marinaki S., Theodora M., Daskalakis G., Frangou E. (2026). Aberrant neutrophil and complement activation in thrombotic microangiopathies in pregnancy—Is there a missing link?. Autoimmun. Rev..

[26] Morelle J., Caravaca-Fontan F., Fakhouri F., Frangou E., Bruchfeld A., Floege J., Mirioglu S., Moran S.M., Steiger S., Stevens K.I. (2025). Complement activation in secondary thrombotic microangiopathies. Nephrol. Dial. Transplant..

[27] Frimat M., Gnemmi V., Stichelbout M., Provôt F., Fakhouri F. (2024). Pregnancy as a susceptible state for thrombotic microangiopathies. Front. Med..

[28] Mirioglu S., Morelle J., Efe O., Hurdogan O., Dirim A.B., Kumru G., Lentine K.L., Caliskan Y. (2026). Thrombotic microangiopathy after kidney transplantation: Diagnosis and management strategies. Clin. Kidney J..

[29] Coscia L.A., Armenti D.P., King R.W., Sifontis N.M., Constantinescu S., Moritz M.J. (2015). Update on the Teratogenicity of Maternal Mycophenolate Mofetil. J. Pediatr. Genet.

[30] (2009). Kidney Disease: Improving Global Outcomes (KDIGO) Transplant Work Group. KDIGO clinical practice guideline for the care of kidney transplant recipients. Am. J. Transpl..

[31] Wagner M., Earley A.K., Webster A.C., Schmid C.H., Balk E.M., Uhlig K. (2015). Mycophenolic acid versus azathioprine as primary immunosuppression for kidney transplant recipients. Cochrane Database Syst. Rev..

[32] Bommer M., Wölfle-Guter M., Bohl S., Kuchenbauer F. (2018). The Differential Diagnosis and Treatment of Thrombotic Microangiopathies. Dtsch. Arztebl. Int..

[33] Mazzierli T., Allegretta F., Maffini E., Allinovi M. (2022). Drug-induced thrombotic microangiopathy: An updated review of causative drugs, pathophysiology, and management. Front. Pharmacol..

[34] Timmermans S., van Paassen P. (2021). The Syndromes of Thrombotic Microangiopathy: A Critical Appraisal on Complement Dysregulation. J. Clin. Med..

[35] Manook M., Kwun J., Sacks S., Dorling A., Mamode N., Knechtle S. (2018). Innate networking: Thrombotic microangiopathy, the activation of coagulation and complement in the sensitized kidney transplant recipient. Transpl. Rev..

[36] Java A., Sparks M.A., Kavanagh D. (2025). Post-transplant Thrombotic Microangiopathy. J. Am. Soc. Nephrol..

[37] Trimarchi H., Coppo R. (2021). Glomerular endothelial activation, C4d deposits and microangiopathy in immunoglobulin A nephropathy. Nephrol. Dial. Transplant..

[38] Maillard N., Wyatt R.J., Julian B.A., Kiryluk K., Gharavi A., Fremeaux-Bacchi V., Novak J. (2015). Current Understanding of the Role of Complement in IgA Nephropathy. J. Am. Soc. Nephrol..

[39] Smith-Jackson K., Harrison R.A. (2023). Alternative pathway activation in pregnancy, a measured amount “complements” a successful pregnancy, too much results in adverse events. Immunol. Rev..

[40] Kavanagh D., Ardissino G., Brocklebank V., Bouwmeester R.N., Bagga A., Ter Heine R., Johnson S., Licht C., Ma A.L.T., Noris M. (2024). Outcomes from the International Society of Nephrology Hemolytic Uremic Syndromes International Forum. Kidney Int..

